# Inhibition of the MEK/ERK pathway suppresses immune overactivation and mitigates TDP-43 toxicity in a *Drosophila* model of ALS

**DOI:** 10.1186/s12979-023-00354-8

**Published:** 2023-06-20

**Authors:** Wenkai Yue, Xue Deng, Zhao Wang, Mingsheng Jiang, Rirong Hu, Yongjia Duan, Qiangqiang Wang, Jihong Cui, Yanshan Fang

**Affiliations:** 1 Interdisciplinary Research Center On Biology and Chemistry, Shanghai Institute of Organic Chemistry, Chinese Academy of Sciences, Shanghai, 201210 China; 2 University of Chinese Academy of Sciences, Beijing, 100049 China

**Keywords:** TDP-43, ALS, MAPK signaling, MEK, ERK, Innate immunity

## Abstract

TDP-43 is an important DNA/RNA-binding protein that is associated with age-related neurodegenerative diseases such as amyotrophic lateral sclerosis (ALS) and frontotemporal dementia (FTD); however, its pathomechanism is not fully understood. In a transgenic RNAi screen using *Drosophila* as a model, we uncovered that knockdown (KD) of *Dsor1* (the *Drosophila* MAPK kinase *dMEK*) suppressed TDP-43 toxicity without altering TDP-43 phosphorylation or protein levels. Further investigation revealed that the *Dsor1* downstream gene *rl* (*dERK*) was abnormally upregulated in TDP-43 flies, and neuronal overexpression of *dERK* induced profound upregulation of antimicrobial peptides (AMPs). We also detected a robust immune overactivation in TDP-43 flies, which could be suppressed by downregulation of the MEK/ERK pathway in TDP-43 fly neurons. Furthermore, neuronal KD of abnormally increased AMPs improved the motor function of TDP-43 flies. On the other hand, neuronal KD of *Dnr1*, a negative regulator of the *Drosophila* immune deficiency (IMD) pathway, activated the innate immunity and boosted AMP expression independent of the regulation by the MEK/ERK pathway, which diminished the mitigating effect of RNAi-*dMEK* on TDP-43 toxicity. Finally, we showed that an FDA-approved MEK inhibitor trametinib markedly suppressed immune overactivation, alleviated motor deficits and prolonged the lifespan of TDP-43 flies, but did not exhibit a lifespan-extending effect in Alzheimer disease (AD) or spinocerebellar ataxia type 3 (SCA3) fly models. Together, our findings suggest an important role of abnormal elevation of the MEK/ERK signaling and innate immunity in TDP-43 pathogenesis and propose trametinib as a potential therapeutic agent for ALS and other TDP-43-related diseases.

**Supplementary Information:**

The online version contains supplementary material available at 10.1186/s12979-023-00354-8.

## Introduction

ALS is an age-related neurodegenerative disease characterized by progressive loss of motor neurons [1]. Protein inclusion containing TAR DNA-binding protein of 43 kDa (TDP-43) is a pathologic hallmark of ALS, and missense mutations in the gene encoding TDP-43 can cause ALS [2–4]. TDP-43 is an important ribonucleoprotein (RNP) that engages in various steps of the regulation of RNA processing and homeostasis [5, 6]. The pathogenesis of TDP-43 has been associated with misregulation of RNAs, abnormal assembly and phase transition of RNP granules such as stress granules and nuclear bodies, impairment of the proteostasis system, dysfunction of mitochondria, etc. [3, 6, 7]. In addition, emerging evidence suggests the involvement of inflammation and innate immunity in TDP-43-mediated neurodegeneration. For example, TDP-43 can activate microglia through the nuclear factor-kappa B (NF-κB) signaling [8, 9] and trigger mitochondrial DNA release to activate neuroimmune and neuroinflammation via the cGAS/STING pathway in ALS [10].

The mitogen-activated protein kinase (MAPK) cascades play a vital role in transduction of extracellular signals and regulation of different cellular functions such as stress and inflammatory responses [11, 12], cell proliferation and differentiation [13, 14], oncogenesis and tumor progression [15], cell death [16], as well as the innate immune signaling [17–19]. Three mammalian MAPK families have been characterized, including the classical MAPKs (also known as extracellular signal-regulated kinases, ERKs), the c-Jun N-terminal kinases (JNKs), and the p38 kinases [20]. And, the involvement of the MAPK signaling pathways in neurological disorders is increasingly recognized. For example, accumulation of phosphorylated MAPK/ERK Kinase (MEK) and abnormal activation of ERK were detected in AD patients [21, 22]; phosphorylated ERK1/2 was found in the central nervous system (CNS) of ALS patients and mouse models [23, 24]; MEK could phosphorylate TDP-43 upon heat shock, which reduced the RNA binding affinity and altered TDP-43-regulated RNA splicing [25]; and inhibition of p38 was reported to suppress TDP-43-induced neurodegeneration in a fly model of ALS [26]. Thus, multiple lines of evidence suggest an important role of the MAPK families in the pathogenesis of ALS and other neurodegenerative diseases.

Immune and inflammatory responses are known to impact on the pathogenesis and progression of several neurological disorders, including ALS, FTD and AD [26–29]. And, chronic activation of microglia and peripheral immune cells in the CNS is a common feature of these diseases [30, 31]. In particular, several recent studies have linked abnormal activation of the MAPK signal pathway in microglia to neurodegeneration. For example, ERK-activated microglia were shown to drive astrogliosis, synaptic loss, neuronal death and neurobehavioral deficits in mice [32], while activation of the NF-κB signaling in microglia induced motor neuron loss in ALS [33]. However, whether a direct, cell-autonomous overactivation of the MAPKs and the innate immunity in neurons play a role in ALS or TDP-43 pathogenesis is yet to be explored.

In this study, we discovered that the *Drosophila* ERK gene *rl* was significantly upregulated in TDP-43 flies, and upregulation of the MEK/ERK pathway in fly neurons induced robust activation of the innate immunity. Moreover, neuron-specific downregulation of the MEK/ERK pathway or KD of the abnormally upregulated AMPs was sufficient to suppress the neurodegenerative phenotypes and extend the shortened lifespan of TDP-43 flies. Finally, feeding flies with trametinib, a small compound inhibitor of MEK, significantly ameliorated TDP-43-induced behavioral deficits in fly models, offering an opportunity for developing new therapeutic strategies aimed at the intervention of ALS and other TDP-43-related diseases.

## Results

### Identification of *Downstream of raf1* (*Dsor1*) and the *Drosophila* MEK/ERK pathway as modifiers of TDP-43 toxicity

**Fig. 1:**
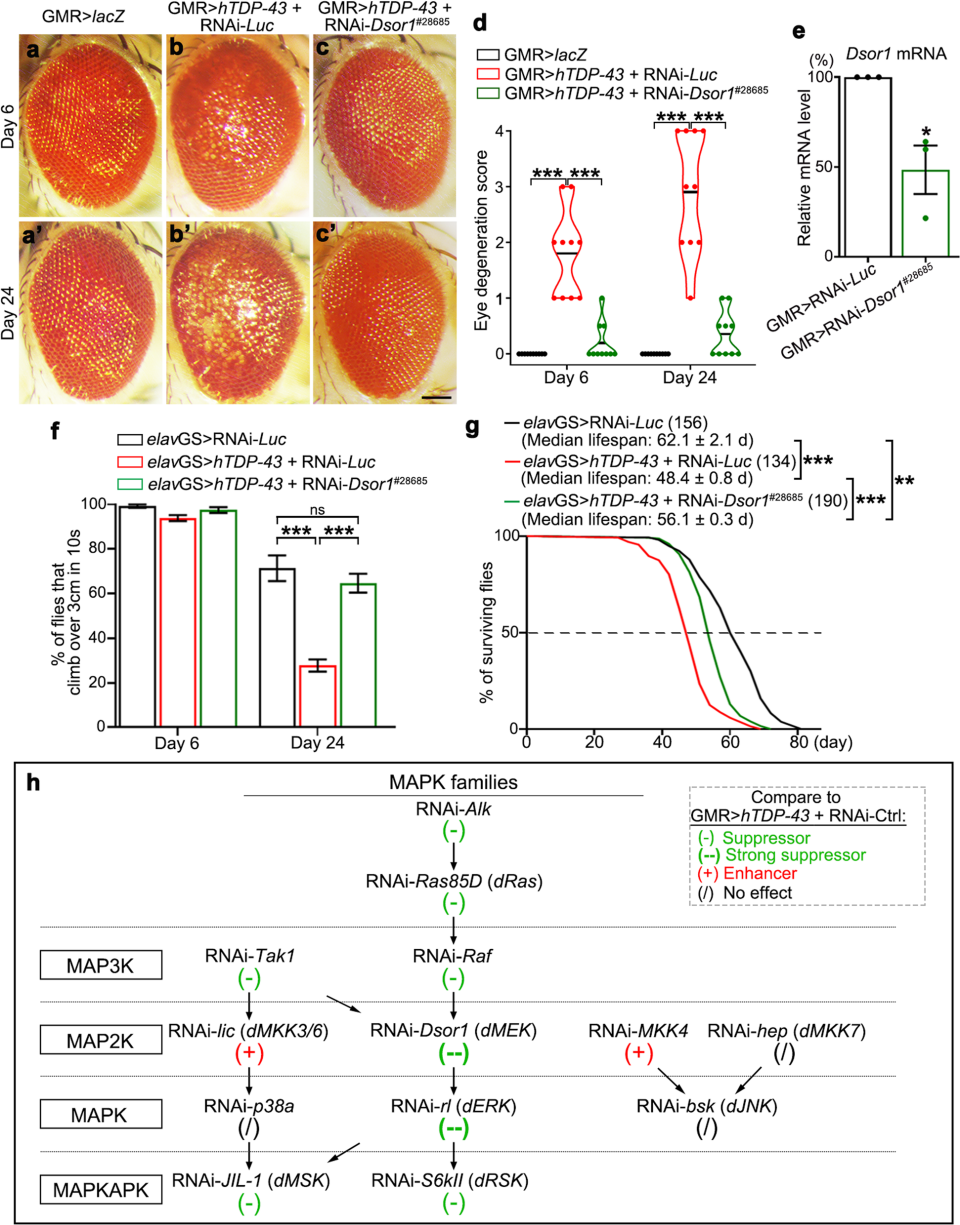
*Dsor1* (*dMEK*) modifies TDP-43-induced cytotoxicity in the in vivo* Drosophila* models. **a-c’** TDP-43-induced eye degeneration (by GMR-Gal4) is suppressed by transgenic RNAi (#28,685) KD of *Dsor1*. UAS-*lacZ* (*lacZ*) and RNAi-*Luciferase* (RNAi-*Luc*) flies are used as the control for UAS-*hTDP-43* and RNAi-*Dsor1*, respectively. **d** The degeneration scores in (**a**-**c**’) are quantified and shown as the violin plots with mean. **e** qPCR analysis confirming the KD of *Dsor1* by RNAi-*Dsor1*. The relative mRNA levels of *Dsor1* are normalized to *actin* and shown as percentage relative to that of the control flies (GMR > RNAi-*Luc*), which is set to 100%. Note that the *Dsor1* mRNA levels are not fully decreased because the RNAi-*Dsor1* transgene is expressed in the fly eye only (with GMR-Gal4), while the mRNA levels are examined in the homogenates of the entire fly head that includes many other cells expressing *Dsor1* but not RNAi-*Dsor1*. **f** Adult-onset neuronal downregulation of *Dsor1* (by *elav*GS) suppresses TDP-43-induced, age-dependent climbing decline. **g** The log-rank analysis of the survival curves shows that KD of *Dsor1* in adult fly neurons extends the shortened lifespan of the *elav*GS > *hTDP-43* flies. The number (n) of flies tested in each group is as indicated; the median lifespan is shown as mean ± SEM and the statistical significance is determined by one-way ANOVA. **h** Summary of the effect of downregulating the fly genes encoding the kinases in the three MAPK families on TDP-43-induced eye degeneration. Mean ± SEM; *n* = 10 eyes/group in (**d**), *n* = 3 in (**e**), *n* =  ~ 10 vials/group with ~ 20 flies each vial in (**f**). One-way ANOVA in (**d**, **f**) and Student’s *t*-test in (**e**). *p < 0.05, **p < 0.01, ***p < 0.001; ns, no significance. Scale bar: 100 μm. See Table S1 for the exact genotypes in each of the fly assays

To reveal unknown genetic modifiers of TDP-43 toxicity, we used the *Drosophila* eye, a well-established and convenient in vivo cytotoxicity model for identifying and investigating new factors that modulate neurodegeneration [34, 35] to carry out a transgenic RNA interference (RNAi) screen. As previously reported [36, 37], expressing human TDP-43 (*hTDP-43*) in fly eyes with a GMR-Gal4 driver (Figure S1a) led to deleterious alterations including rough eye surface, loss of pigment cells, eye swelling and deformation (Figure S1b and Fig. 1a-b’). Since TDP-43 pathology is often associated with hyperphosphorylation [38, 39] and the phosphorylation state of TDP-43 protein is positively correlated with its toxicity [40–42], we focused on the fly genes encoding protein kinases and phosphatases in one set of the transgenic RNAi screen. Among them, we found that two independent RNAi lines (#28,685 and #31,184) of the gene *Dsor1*, the *Drosophila* homologue of mammalian MEK (*dMEK*), showed dramatic suppression of the age-dependent eye degeneration of the TDP-43 flies (Fig. 1b-d and Figure S2a-c). The KD efficiency of RNAi-*Dsor1* was examined and confirmed by real-time quantitative PCR (qPCR) analysis (Fig. 1e and Figure S2d).

The clinical symptoms of ALS are characterized by loss of control of voluntary muscle movements such as walking and talking due to gradual deterioration of the motor neurons [1], and the median survival time of the ALS patients from onset to death ranges from 20 to 48 months [43]. In order to further evaluate the modifier genes in a more ALS-relevant system, we expressed *hTDP-43* in adult fly neurons with an inducible pan-neuronal *elav*-GeneSwitch (GS) driver, which led to age-dependent climbing decline (Fig. 1f) and shortened lifespan (Fig. 1g). Neuronal KD of *Dsor1* by either of the UAS-RNAi lines significantly suppressed TDP-43-induced climbing defects (Fig. 1f and Figure S2e) and markedly extended the shortened lifespan of the TDP-43 flies (Fig. 1g and Figure S2f). Since the two RNAi-*Dsor1* lines exhibited similar modifying effects, for simplicity, #28,685 was designated as the “RNAi-*Dsor1*” line and used in the rest of the study.

In addition to *Dsor1*, the effects of RNAi KD of other fly genes encoding MAPK kinases on TDP-43-induced eye degeneration were summarized in Fig. 1h. The results showed that downregulation of the MEK/ERK pathway, including the genes such as *Alk*, *Ras85D* (*dRas*), *Raf, Dsor1* (*dMEK*), *rl* (*dERK*) and *S6kII* (*dRSK*), significantly suppressed the eye degeneration of the TDP-43 flies. Although previously reported to modify TDP-43 toxicity [26], downregulation of the p38 or the JNK pathway did not show consistent or robust suppression in our study. Rather, in the cases of *lic* (*dMKK3/6*) and *MKK4*, their downregulation enhanced the toxicity of TDP-43. Thus, these data suggested that, of the three main MAPK families, the MEK/ERK pathway was closely involved in TDP-43-mediated neurodegeneration.

## Abnormal upregulation of *rl* and the MEK/ERK pathway in TDP-43 flies

**Fig. 2:**
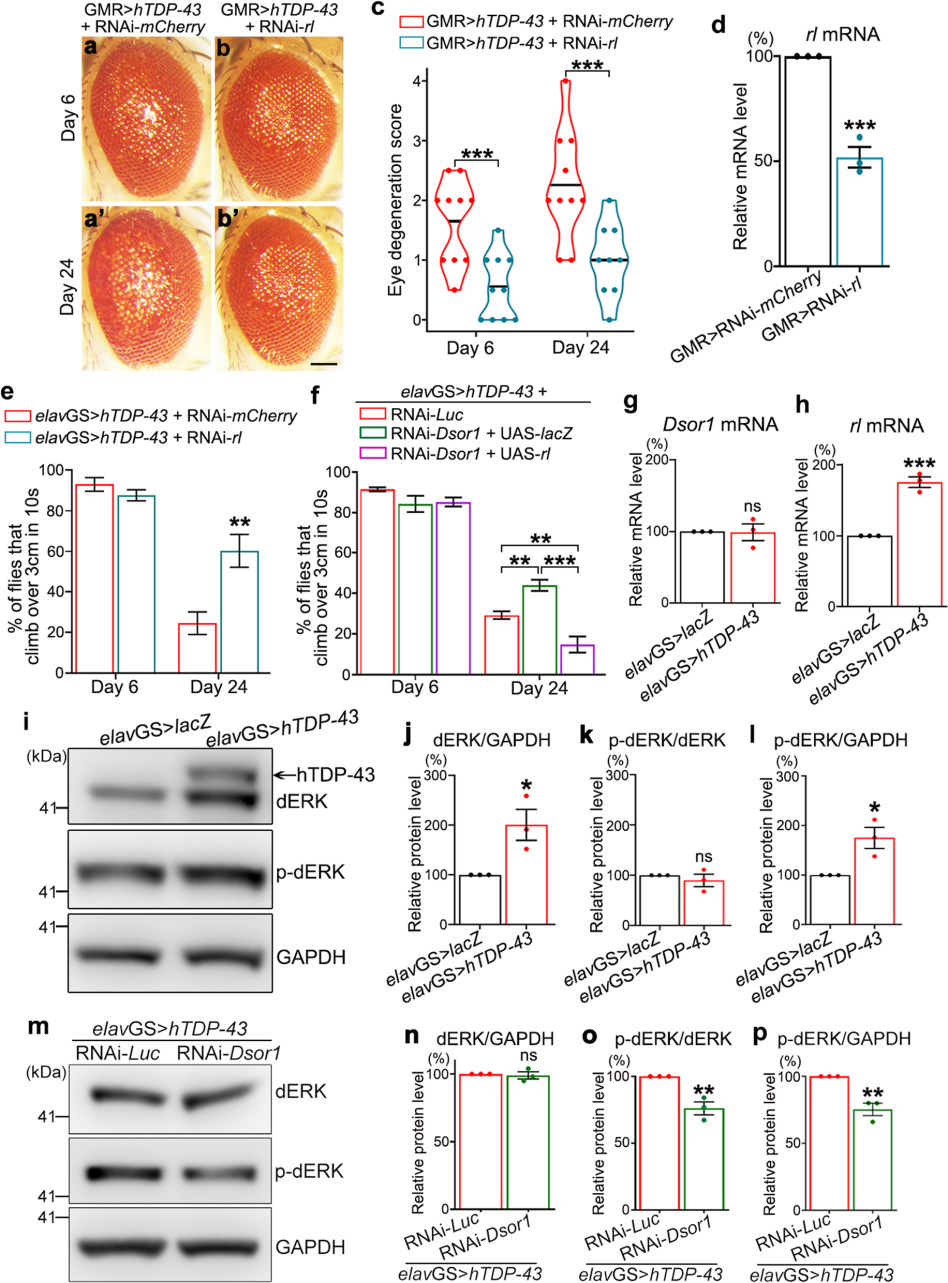
*rl* (*dERK*) is upregulated in TDP-43 flies and downregulation of the fly MEK/ERK pathway suppresses TDP-43 toxicity. **a-b’** TDP-43-induced eye degeneration (by GMR-Gal4) is suppressed by transgenic RNAi KD of *rl*. RNAi-*mCherry* flies are used as the control for RNAi-*rl*. **c** The degeneration scores in (**a**-**b**’) are quantified and shown as violin plots with mean. **d** qPCR analysis of the KD efficiency of RNAi-*rl*. Note that the *rl* mRNA levels are not fully decreased because the RNAi-*rl* transgene is expressed in the fly eye only (with GMR-Gal4), while the mRNA levels are examined in the homogenates of the entire fly head that includes many other cells expressing *rl* but not RNAi-*rl*. **e** Adult-onset neuronal downregulation of *rl* (by *elav*GS) suppresses TDP-43-induced, age-dependent climbing decline. **f** Neuronal upregulation of *rl* abolishes the suppressive effect of RNAi-*Dsor1* (*dMEK*) on TDP-43-induced climbing defects. **g**-**h** qPCR analysis of the mRNA levels of *Dsor1* (**g**) and *rl* (**h**) in TDP-43 fly heads. All mRNA levels are normalized to *actin* and shown as percentages to that of the respective control flies (which is set to 100%): RNAi-*mCherry* in (**d**) and UAS-*lacZ* in (**g**-**h**). **i**-**p** Representative western blot images (**i**, **m**) and quantifications of the relative dERK protein levels (**j**, **n**) as well as phosphorylated dERK (p-dERK) levels normalized to total dERK (**k, o**) or GAPDH (**l**, **p**) in the “*elav*GS > *hTDP-43*” (**i**-**l**) or the “*elav*GS > *hTDP-43* + RNAi-*Dsor1*” (**m**-**p**) fly heads are shown. Of note, because the anti-ERK and the anti-pERK antibodies are both of the rabbit origin, for western blotting of ERK and pERK in this study, equal amounts of the same samples were examined on two parallel gels. Mean ± SEM; *n* = 10 eyes/group in (**c**), *n* = 3 in (d, g-h, j-l, n-p), and *n* =  ~ 10 vials/group with ~ 20 flies each vial in (**e**–**f**). Student’s *t*-test except for one-way ANOVA in (**f**). *p < 0.05, **p < 0.01, ***p < 0.001; ns, no significance. Scale bar: 100 μm

Among the tested genes encoding kinases of the fly MEK/ERK pathway, KD of *rl*, the *Dsor1* downstream gene encoding the *Drosophila* ERK (dERK), showed the second strongest suppression of TDP-43-induced eye degeneration (Fig. 2a-d). And, neuronal KD of *rl* substantially improved the motor function of the *elav*GS > *hTDP-43* flies in the climbing assay (Fig. 2e). Further, to confirm that *rl* was genetically downstream of *Dsor1* in the effect of modifying TDP-43 toxicity, we constructed a stable fly stain that expressed both *hTDP-43* and RNAi-*Dsor1* in fly neurons (“*elav*GS > *hTDP-43,* RNAi-*Dsor1*”; see Methods and Table S1). We then upregulated *rl* in the neurons of these flies, which indeed abolished the mitigating effect of RNAi-*Dsor1* on TDP-43-induced climbing defects (Fig. 2f).

Hyperphosphorylation of TDP-43 at serine 409 and 410 (pTDP-43) is a disease-hallmarked change of TDP-43 protein in ALS and FTD [38, 39]. Next, we examined whether KD of *Dsor1* or *rl* affected the protein abundance or phosphorylation levels of the transgenically expressed hTDP-43 in the heads of the *elav*GS > *hTDP-43* flies. The western blot results indicated that hTDP-43 protein levels were unaffected by neuronal KD of *Dsor1* or *rl* (Figure S3a, b, e, f). To our surprise, neither the phosphorylation levels (ratio of pTDP-43 to total TDP-43) nor the abundance of pTDP-43 proteins (normalized to GAPDH) was significantly altered by RNAi-*Dsor1* (Figure S3c, d) or RNAi-*rl* (Figure S3g, h). In addition, transgenically expressed wild-type (WT) hTDP-43 was soluble and did not result in insoluble aggregation in fly models (Figure S3i; and [42, 44, 45]), which excluded the possibility that the mitigating effect by downregulation of *Dsor1* or *rl* was due to reducing hTDP-43 protein aggregates.

Since KD of *Dsor1* or *rl* in fly neurons did not show a significant impact on TDP-43 phosphorylation, it raised an alternative hypothesis that the MEK/ERK pathway was misregulated in TDP-43 flies, which contributed to TDP-43-induced neurodegeneration and therefore could be rescued by RNAi-*Dsor1* or RNAi-*rl*. Indeed, although the mRNA levels of *Dsor1* were unchanged by TDP-43 (Fig. 2g), *rl* expression was significantly increased in the TDP-43 fly heads, both at the mRNA (Fig. 2h) and the protein levels (Fig. 2i, j). As a result, although the phosphorylation levels of dERK were not increased (Fig. 2k), phosphorylated dERK (p-dERK) protein was significantly more abundant in the heads of the *elav*GS > *hTDP-43* flies (Fig. 2l). Further, we confirmed that neuronal KD of *Dsor1* decreased the phosphorylation levels of dERK (Fig. 2m-p), suggesting that the suppression of TDP-43-induced toxicity by RNA-*Dsor1* was attributed to the reduction of phosphorylated dERK. Together, these results indicated that *rl* and the MEK/ERK pathway were abnormally upregulated in the TDP-43 flies, which may underlie the TDP-43-induced cytotoxicity.

## MEK/ERK-mediated immune overactivation contributes to TDP-43 toxicity

**Fig. 3:**
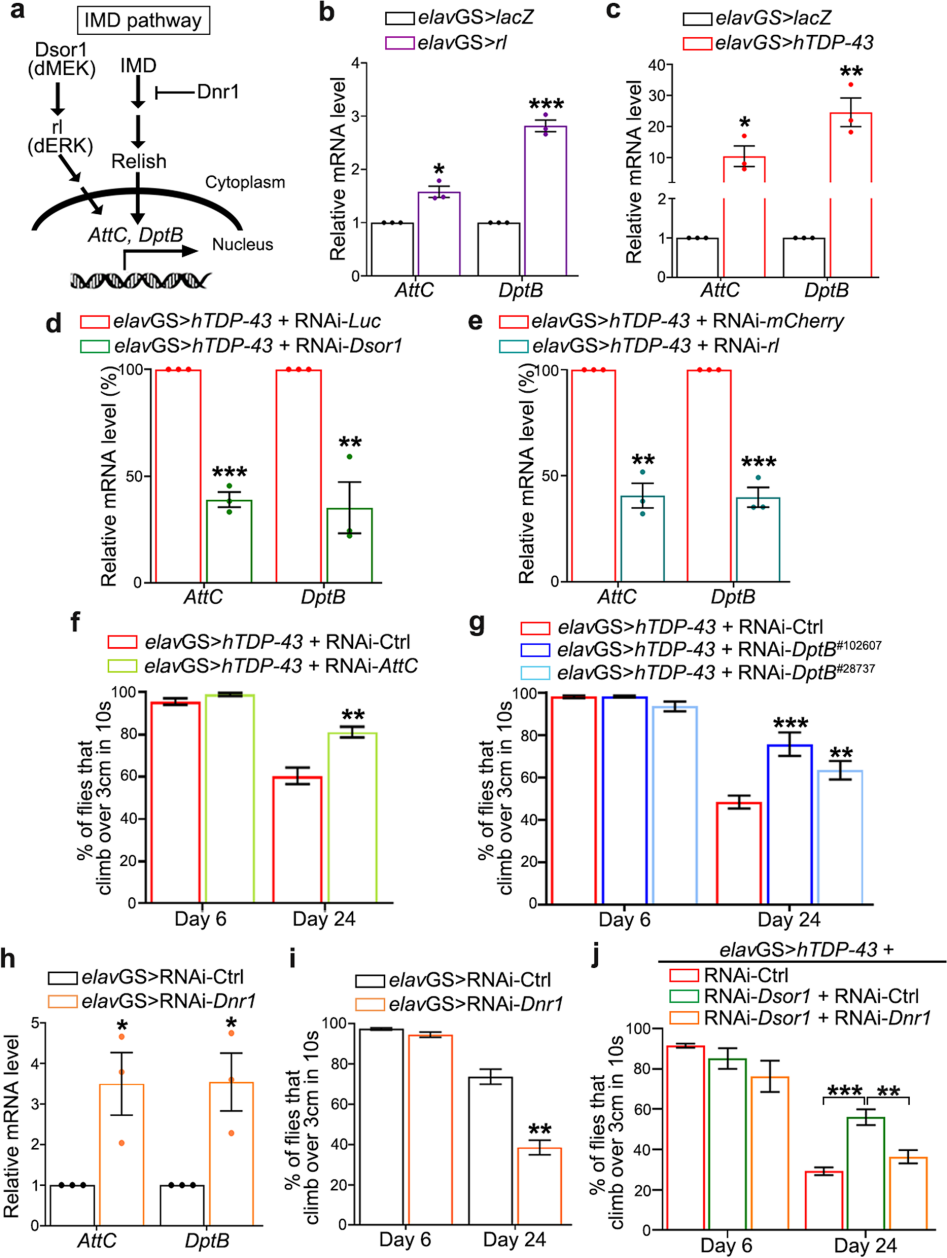
MEK/ERK-mediated immune overactivation contributes to TDP-43 pathogenesis. **a** A schematic of the MEK/ERK signaling and the IMD immune pathway. **b**-**e** qPCR analysis of the relative mRNA levels of the immune AMPs *AttC* and *DptB* in fly heads of the indicated genotypes (also see Table S1). **f**-**g** Adult-onset neuronal KD of *AttC* (**f**) or *DptB* (**g**) suppresses TDP-43-induced, age-dependent climbing decline. **h**-**j** Neuronal KD of *Dnr1* significantly increases immune AMP expression (**h**), causes age-dependent motor deficits (**i**), and abolishes the suppressive effect of RNAi-*Dsor1* on TDP-43-induced climbing defects (**j**). All mRNA levels are normalized to *actin* and shown as fold change (**b**, **c**, **h**) or percentage (**d**-**e**) to that of the corresponding control flies (which is set to 1 or 100%): UAS-*lacZ* in (**b-c**), RNAi-*Luc* in (**d**), RNAi-*mCherry* in (**e**), and RNAi- Ctrl^V^.^60200^ flies in (**h**) (also see Methods and Table S1). Mean ± SEM; *n* = 3 in (**b**-**e**, **h**), and *n* =  ~ 10 vials/group with ~ 20 flies each vial in (**f**, **g**, **I**, **j**). Student’s *t*-test in (**b**-**f**, **h**, **i**) and one-way ANOVA in (**g**, **j**). *p < 0.05, **p < 0.01, ***p < 0.001

The MAPK signaling cascade transmits cell signals and regulates gene expression in a variety of cellular events and functions including innate immunity [19, 46]. The transcription factors downstream of the MAPK pathways such as Fos and Jun play a pivotal role in the expression of cytokines and other genes critical for immune responses [47, 48]. In *Drosophila*, the orthologue of Jun, Jra, is a core element of the immune gene regulatory network and fine-tune the immune responses including the expression of many AMPs in the fly gut [49]. Meanwhile, deregulation of TDP-43 was shown to activate NF-κB and promote immune responses in ALS patients and TDP-43 transgenic mice [8, 10]. Together, these previous observations raised the possibility that downregulation of the MEK/ERK pathway with RNAi-*Dsor1* or RNAi-*rl* suppressed TDP-43-induced toxicity by reducing immune overactivation (Fig. 3a).

Consistent with this hypothesis, we found that the fly MEK/ERK pathway tightly regulated the expression levels of AMPs. For example, neuronal upregulation of *rl* (Fig. 3b) increased whereas downregulation of *Dsor1* (Figure S4a) decreased the mRNA levels of *AttC* and *DptB* in fly heads. Further, we examined the mRNA levels of multiple AMPs in the brains of the *elav*GS > *hTDP-43* flies, including *AttC*, *DptB*, *AttA*, *DptA* and *Dro* of the IMD pathway, *Drs* of the Toll pathway, and *Mtk* in both pathways [50–52]. All of these AMPs were dramatically upregulated (Fig. 3c and Figure S4b), indicative of a profound immune overactivation [53, 54]. More importantly, we showed that KD of *Dsor1* or *rl* in fly neurons potently decreased the expression of *AttC* and *DptB* in the brain of the *elav*GS > *hTDP-43* flies (Fig. 3d, e). And, among the aberrantly upregulated AMP genes in the TDP-43 flies, neuronal KD of merely a single AMP gene, e.g., either *AttC* or *DptB* (Figure S5a, b), was sufficient to markedly reduce TDP-43-induced, age-dependent climbing decline (Fig. 3f, g).

As mentioned earlier, the MEK/ERK pathway participates in a variety of different cellular functions and regulations [14, 55]. To determine whether the immune suppression played a major role in the mitigating effect of downregulation of the MEK/ERK pathway, we sought for an independent pathway to activate the innate immunity in the “*elav*GS > *hTDP-43*, RNAi-*Dsor1*” flies and to test whether and how much RNAi-*Dsor1* could still mitigate TDP-43 toxicity. *Defense repressor 1* (*Dnr1)* was reported to be a potent negative regulator of the IMD immune pathway in flies [56]. Indeed, both ubiquitous (Figure S5c) and neuron-specific (Fig. 3h) KD of *Dnr1* in adult flies led to substantial upregulation of immune AMPs as well as age-dependent decline of the motor function (Fig. 3i). Moreover, inducing immune overactivation by KD of *Dnr1* in the neurons of the “*elav*GS > *hTDP-43*, RNAi-*Dsor1*” flies almost completely abolished the suppression of RNAi-*Dsor1* on TDP-43 toxicity in the climbing assay (Fig. 3j).

Together, these results indicated that abnormal elevation of the MEK/ERK signaling led to immune overactivation that contributed a significant portion to TDP-43-induced cytotoxicity, and genetic downregulation of the MEK/ERK pathway in fly neurons was sufficient to suppress immune overactivation and TDP-43 pathogenesis.

## The MEK inhibitor (MEKi) trametinib ameliorates TDP-43-induced immune overactivation and behavioral phenotypes in flies

**Fig. 4:**
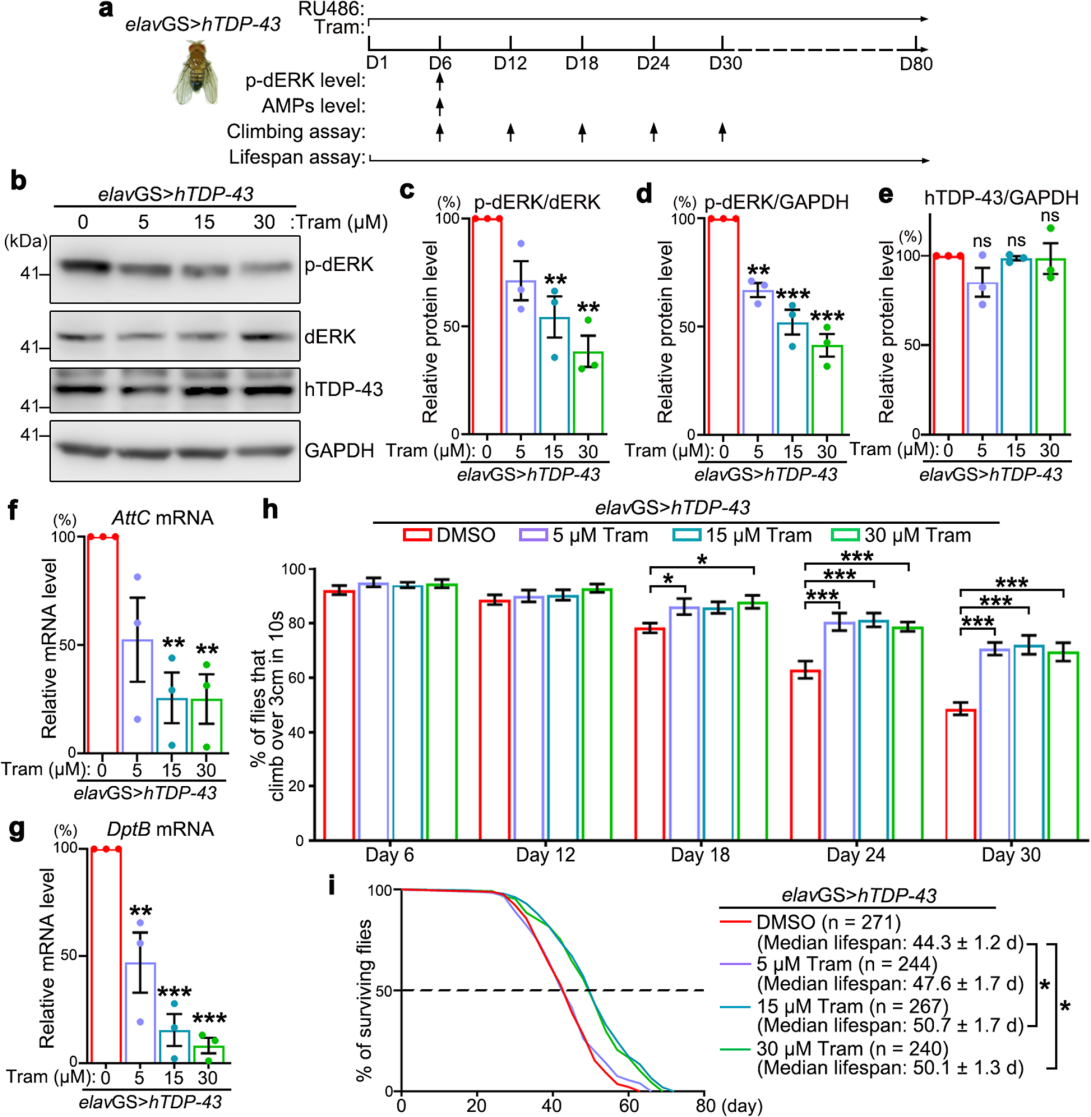
The MEKi trametinib improves the motor function and extends the lifespan of the TDP-43 flies. **A** A schematic of the pharmacologic and behavioral tests of the MEKi trametinib. The expression of the UAS-*hTDP-43* transgene (by *elav*GS) is induced on Day 1 of the adulthood of the flies with RU486. At the same time, trametinib at indicated concentrations is also added in the fly food. **b**-**e** Representative western blot image (**b**) and quantifications of phosphorylated dERK (p-dERK) normalized to total dERK (**c**) or to GAPDH (**d**) and hTDP-43 protein levels (**e**) in the brains of the *elav*GS > *hTDP-43* flies. (**f**, **g**) qPCR analysis of the mRNA levels of the immune AMP genes *AttC* (**f**) and *DptB* (**g**) in the brain of the TDP-43 flies fed with indicated concentrations of trametinib. The mRNA levels are normalized to *actin* and shown as percentages to that of the control flies (*elav*GS > *hTDP-43* + DMSO), which is set to 100%. **h** The climbing assays of the *elav*GS > *hTDP-43* flies fed with indicated concentrations of trametinib. **i** The log-rank analysis of the survival curves indicates that trametinib extends the lifespan of the *elav*GS > *hTDP-43* flies. Mean ± SEM; *n* = 3 in (**c**-**g**), *n* =  ~ 10 vials/group with ~ 20 flies each vial in (**h**), and the number (n) of flies tested in each group is as indicated in (i). One-way ANOVA; *p < 0.05, **p < 0.01, ***p < 0.001; ns, no significance

Finally, as an attempt to assess the therapeutic potential of MEKi in treating TDP-43-assoicated ALS, we examined the effectiveness of an FDA-approved MEKi trametinib using the *elav*GS > *hTDP-43* flies (Fig. 4a). Expression of *hTDP-43* was induced in adult fly neurons from Day 1 together with DMSO or trametinib of indicated concentrations added in the fly food. The phosphorylation and protein levels of dERK were examined on Day 6, and indeed dERK phosphorylation was significantly decreased and displayed a dosage-dependent manner (Fig. 4b-d). Meanwhile, the protein levels of transgenically expressed hTDP-43 were not decreased, indicating that the addition of different concentrations of trametinib did not significantly affect the uptake of fly food with RU486 or the induction of hTDP-43 expression (Fig. 4b, e)*.*

Consistent with KD of *Dsor1* in the earlier genetic experiments, pharmacological inhibition of Dsor1 by the MEKi trametinib significantly reduced the mRNA levels of the AMPs *AttC* and *DptB* in TDP-43 fly heads (Fig. 4f, g). More importantly, we performed the climbing and lifespan assays to evaluate the effect of trametinib on the motor function and longevity of TDP-43 flies and the results indicated that 5 μM of trametinib was sufficient to suppress TDP-43-induced motor deficits (Fig. 4h) and 15 μM of trametinib could extend the shortened lifespan of the *elav*GS > *hTDP-43* flies (Fig. 4i), while higher concentrations did not further increase the mitigating effects. Immune responses are associated with aging and neurodegenerative diseases, and an earlier studied reported that treatment of the MEK inhibitor trametinib increased the longevity of WT flies [57]. To examine whether inhibition of the MEK/ERK pathway could manifest a general beneficial effect in other disease models, we test the effect of trametinib in an AD model expressing *Aβarc* [28, 29] and a polyglutamine (polyQ)-mediated SCA3 model expressing *SCA3-Q84* in fly neurons [58]. However, the MEKi trametinib did not extend but rather showed a tendency to further shorten the lifespan of the AD or SCA3 fly models (Figure S6). Together, inhibition of the MEK/ERK pathway showed a specific mitigating effect to TDP-43 toxicity, which was consistent with the abnormal upregulation of *dERK* levels and the MEK/ERK pathway in hTDP-43 flies.

## Discussion

**Fig. 5:**
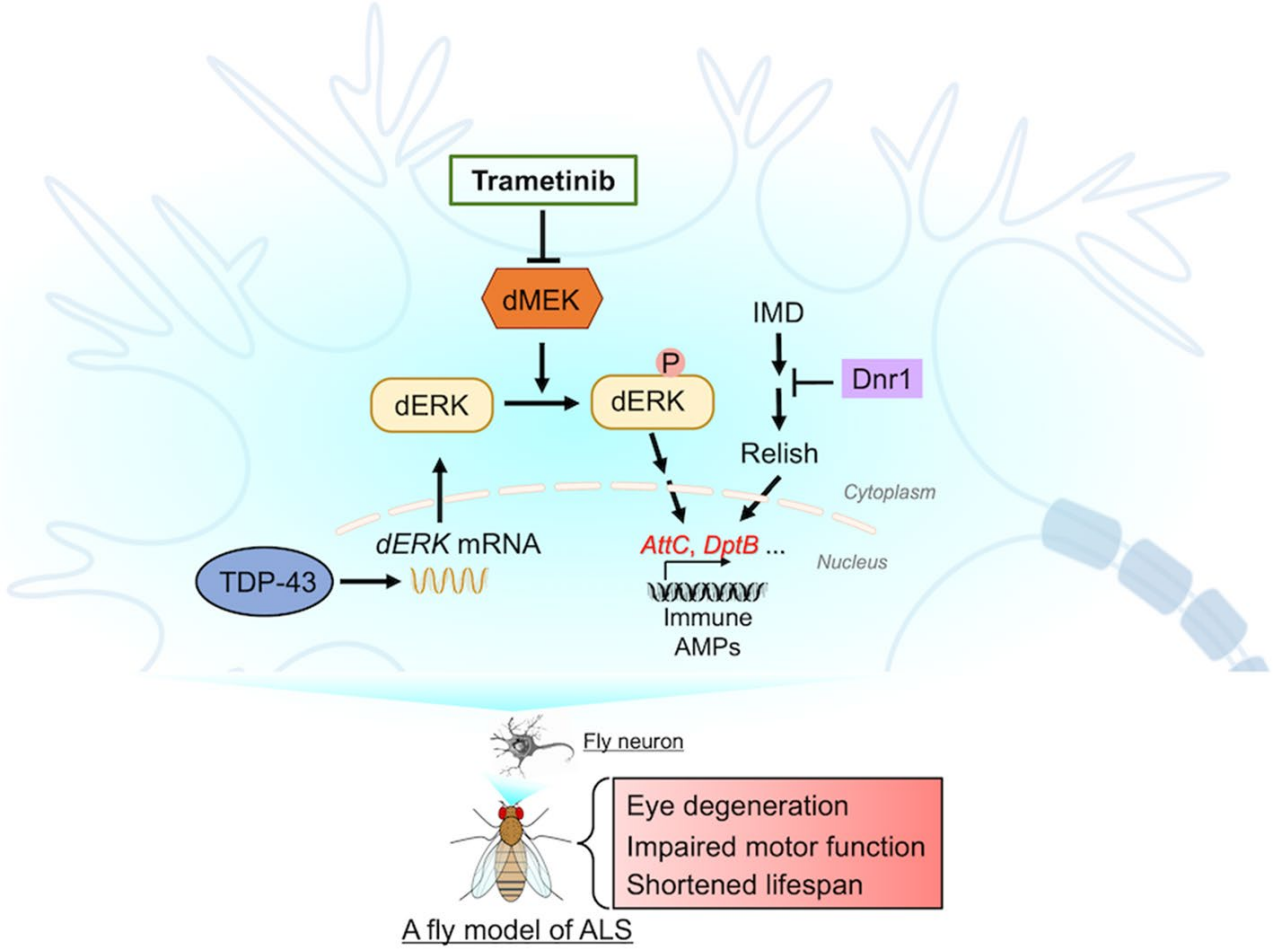
A schematic model of abnormal elevation of the MEK/ERK pathway and immune overactivation in TDP-43-induced cytotoxicity. In this study, we uncover that *dERK* (*rl*) and the MEK/ERK pathway is abnormally upregulated in TDP-43 flies, which induces immune overactivation. Neuron-specific downregulation of the MEK/ERK signaling or the immune AMPs such as *AttC* and *DptB* significantly suppresses TDP-43 toxicity. Moreover, the MEKi trametinib ameliorates TDP-43-induced degenerative phenotypes and extends the lifespan in flies. Together, MEKi may be a potential target for developing new therapeutic strategies to treat ALS and related diseases

In this study, we identified RNAi-*Dsor1* (*dMEK*) as a genetic suppressor of TDP-43 toxicity in a fly screen. Further examination uncovered that transgenic expression of *hTDP-43* caused a remarkable upregulation of the fly ERK gene *rl*, leading to the aberrant elevation of the MEK/ERK pathway in TDP-43 flies. Upregulation of the MEK/ERK pathway in fly neurons activated the innate immunity, evidenced by a dramatic increase in the expression of the immune AMP genes, which was also observed in the *elav*GS > *hTDP-43* fly brains. More importantly, we showed that KD of the upregulated AMPs such as *AttC* and *DptB* suppressed TDP-43-induced motor deficits, whereas immune overactivation by KD of *Dnr1*, a negative regulator of the IMD pathway, abolished the mitigating effect of RNAi-*Dsor1* on TDP-43 toxicity (Fig. 5).

Abnormal TDP-43 protein inclusions in the patients with ALS or FTD are often associated with TDP-43 hyperphosphorylation and phosphorylation levels are positively correlated with TDP-43 toxicity [38, 39, 59, 60]. Several kinases phosphorylating TDP-43 have been discovered, including the casein kinase 1 family, tau tubulin kinases 1 and 2, and cell division cycle kinase 7, and inhibition of these kinases could mitigate TDP-43-mediated deleterious effects in cell and animal models [40–42, 61–65]. In this study, we identified the MAPK family kinase Dosr1 (dMEK) as a modifier of TDP-43 toxicity; however, manipulation of dMEK or its downstream kinase rl (dERK) levels did not significantly alter TDP-43 phosphorylation levels. Rather, our investigation indicated that TDP-43 regulated and acted upstream of the MEK/ERK pathway, as the mRNA and protein levels of *dERK* were markedly increased in TDP-43 flies. And, we further showed that the abnormal elevation of the MEK/ERK pathway led to overactivation of the innate immunity, which contributed to TDP-43 pathogenesis.

TDP-43 could activate inflammatory and immune responses via the cGAS/STING pathway by triggering mitochondrial DNA release in neurons [10]. Nevertheless, the involvement of the MEK/ERK signaling in the regulation of inflammation and immunity was reported mostly in microglia and other immune-related cells [32, 66]. Here, we demonstrated that TDP-43 can activate the innate immunity by upregulating the MEK/ERK pathway in fly neurons. The role of immunity and inflammation in neurodegeneration has been double-edged. On one hand, activated immunity accelerates the clearance of protein aggregates and damaged cells [67]; on the other hand, prolonged immune overactivation releases excessive cytokines that cause neuronal cell death and additional deleterious effects that exacerbate neurodegeneration [54, 68].

It should be noted that, all the genetic manipulations in this study, including transgenic expression of *hTDP-43*, downregulation of the MEK/ERK pathway, and genetic inhibition or overactivation of the innate immunity, were restricted to mature neurons in flies. In other words, if the immune responses were entirely secondary to TDP-43-induced neurodegeneration or only involved glia, neuronal KD of the immune AMPs would not have been able to rescue TDP-43 flies. Nevertheless, our data do not exclude the possibility that the initial cell-autonomous immune overactivation in neurons triggers a subsequent avalanche of immune responses from outside neurons, which together lead to the overall degenerative consequences. Collectively, our data indicate that the abnormal elevation of the MEK/ERK signaling promotes the innate immunity in fly neurons, which contributes to TDP-43 toxicity.

The Ras/Raf/MEK/ERK cascade is an important multi-function signaling pathway and plays a critical role in tumorigenesis. The MEKi hence have been developed for treating cancers such as melanoma and non-small cell lung cancer [69–71]. Trametinib was the first FDA-approved MEKi for treatment of melanoma. In this study, we used the in vivo fly model of ALS to assess the effectiveness of the MEKi trametinib, which substantially suppressed TDP-43-mediated immune overactivation and markedly mitigated TDP-43-induced motor deficits. In particular, feeding flies with trametinib could extend the median lifespan of TDP-43 flies by 14.4%, equivalent to roughly 10.2 ~ 10.8 years for humans (WorldData.info). Nevertheless, future research is warranted to confirm the efficacy of the MEKi on other animal models as well as to further dissect the pathogenic mechanism of the “TDP-43 > MEK/ERK pathway > innate immunity” axis. In addition, we notice that a phase I/II clinical trial to evaluate the safety and efficacy of trametinib for treatment of ALS has been filed (ClinicalTrials.gov Identifier NCT04326283). Therefore, it is reasonable to have high hopes for the MEKi trametinib as a potential therapeutic agent for developing new drugs to treat ALS and other TDP-43-related neurodegenerative diseases.

## Supplementary Information

**Additional file 1.**

**Additional file 2.**

## Data Availability

All data generated during this study are included in this published article and its supplementary information files. All unique reagents and materials generated in this study are available from the corresponding author on reasonable request with a material transfer agreement (MTA).

## Abbreviations

AD: Alzheimer disease
ALS: Amyotrophic lateral sclerosis
AMP: Antimicrobial peptide
CNS: Central nervous system
dERK: *Drosophila* ERK
dMEK: *Drosophila* MEK
dMKK3/6: *Drosophila* MKK3/6
Dnr1: Defense repressor 1
dRas: *Drosophila* Ras
dRSK: *Drosophila* RSK
Dsor1: Downstream of raf1
ERK: Extracellular signal-regulated kinase
FTD: Frontotemporal dementia
GS: GeneSwitch
hTDP-43: Human TDP-43
IMD: Immune deficiency
JNK: C-Jun N-terminal kinase
KD: Knockdown
MAPK: Mitogen-activated protein kinase
MEK: MAPK/ERK Kinase
MEKi: MEK inhibitor
NF-κB: Nuclear factor-kappa B
p-dERK: Phosphorylated dERK
polyQ: Polyglutamine
pTDP-43: Hyperphosphorylation of TDP-43 at serine 409 and 410
qPCR: Real-time quantitative PCR
RNAi: RNA interference
RNP: Ribonucleoprotein
SCA3: Spinocerebellar ataxia type 3
TDP-43: TAR DNA-binding protein of 43 kDa
WT: Wild-type
