# Supplementary material for: Inhibition of the MEK/ERK pathway suppresses immune overactivation and mitigates TDP-43 toxicity in a Drosophila model of ALS
Source: Immun Ageing. 2023 Jun 20;20:27. doi: 10.1186/s12979-023-00354-8 (PMC10280928; doi:10.1186/s12979-023-00354-8)
Supplement: Supplementary file 1 — Additional file 1. [file 12979_2023_354_MOESM1_ESM.pdf]

SUPPLEMENTARY INFORMATION

Inhibition of the MEK/ERK pathway suppresses immune overactivation and mitigates TDP-43 toxicity in a *Drosophila* model of ALS

Wenkai Yue, Xue Deng, Zhao Wang, Mingsheng Jiang, Rirong Hu, Yongjia Duan, Qiangqiang Wang, Jihong Cui, and Yanshan Fang

Correspondence to: fangys@sioc.ac.cn

Supplementary Inventory

1. Materials and Methods

2. Supplementary References (1-2)

3. Supplementary Figures (1-6)

4. Supplementary Table (1)

MATERIALS AND METHODS

Drosophila strains

The following fly strains were obtained from the Bloomington Drosophila Stock Center (BDSC), UAS-*lacZ* (#8529), UAS-*rl* (#36270), GMR-Gal4 (#84247), RNAi-*mCherry* (#35785, a control for short hairpin RNAi knockdown), RNAi-*luciferase* (#31603, a control for long hairpin RNAi knockdown), *elav*GS (#43642), RNAi-*Dsor1* (#28685, #31184), UAS-*SCA3-Q84* (#33610). The followings were from the Vienna *Drosophila* Resource Center (VDRC), Austria: RNAi-control (#60200), RNAi-*Dnr1* (#20239), RNAi-*DptB* (#102607, #28737), RNAi-*AttC* (#101213). The followings were from the Tsinghua Fly Center (THFC), China: RNAi-*Alk* (THU2680), RNAi-*Ras85D* (THU1525), RNAi-*Tak1* (THU5798), RNAi-*Raf* (THU5796), RNAi-*lic* (THU5788), RNAi-*MKK4* (THU5416), RNAi-*hep* (THU4047), RNAi-*rl* (THU0191), RNAi-*p38a* (THU2638), RNAi-*bsk* (THU1918), RNAi-*JIL-1* (THU4961), RNAi-*S6kII* (THU2819).

For neuronal expression of long hairpin RNAi lines used in this study, a copy of UAS-*Dcr2* was co-expressed to boost the knockdown efficiency [1]. And, for the various RNAi flies from different sources examined in this study, the mRNA and protein levels as well as the behavioral assays are always compared to their respective backcrossed isogenized RNAi control lines as specified in each experiment. The UAS-*hTDP-43* flies were described previously [2], and the *Tub*GS and the UAS-*Aβarc* fly lines were the kind gifts from Dr. N. Bonini and Dr. D. Huang, respectively.

For simultaneous and independent genetic manipulations, the following stable fly lines carrying multiple transgenes were generated:

w^{1118}; UAS-*hTDP-43*/Cyo; *elav*GS/TM6B.Tb

w^{1118}; UAS-*hTDP-43*/Cyo; *elav*GS,UAS-*Dcr2*/TM6B.Tb

w^{1118}; UAS-*hTDP-43*/Cyo; GMR-gal4/TM6B.Tb

w^{1118}; UAS-*hTDP-43*/Cyo; *elav*GS,UAS-*Dcr2*,RNAi-*Dsor1*/TM6B.Tb

All flies were raised on standard cornmeal media and maintained at 25°C and 60% relative humidity. The specific genotypes of the flies used in each assay in the figures are summarized

in Table S1. Unless otherwise noted, only male flies were tested in the age-associated behavioral assays. For adult-onset, neuronal expression of the UAS-*hTDP-43* and other UAS-RNAi transgenes, the *elav*GS and *Tub*GS driver were induced by supplementing the regular fly food with 80 µg/mL RU486 (TCI, 84371-65-3). For the *in vivo* MEK inhibitor tests, DMSO or Trametinib (Selleck, S2673) was added in the fly food at the concentrations specified in the main text.

Fly eye degeneration assessment

To evaluate the integrity of the *Drosophila* eye, z-stack images of the external eyes of adult flies at indicated ages were acquired using an Olympus SZX16 stereomicroscope. The severity of the eye degeneration was assessed by rough surface, swelling and loss of pigment cells of the compound eyes. Each fly eye was single-blindly scored in a scale of 0 to 4, with 0 for no degeneration and 4 for the complete degeneration. See also Figure S1.

Climbing assay

For the climbing assay, 15-20 flies were transferred into an empty polystyrene vial and gently tapped down to the bottom of the vial. The number of flies that climbed over a distance of 3 cm within 10 seconds was recorded. The test was repeated three times for each vial and about 10 vials per group were examined.

Lifespan assay

For the lifespan experiments, 20 flies per vial and about 10 vials per group were tested. Flies were transferred to fresh fly food every 3 days and the number of dead flies in each vial was recorded. The log-rank test was used for analyzing the lifespan curves and the “50% survival” shown on the curves was derived from the compilation of all vials of the same group. The actual median lifespan of each group was calculated as the average age (day) at which 50% of the flies in a vial died. The statistical significance of the median lifespans between two or more groups was determined by Student’s *t*-test or one-way analysis of variance (ANOVA),

respectively. Flies lost prior to natural death because of escape or accidental death were excluded from the final analysis.

Protein extraction and western blotting

Fly heads or dissected fly brains were homogenized and lysed in ice cold 2% SDS lysis buffer (100 mM Tris-HCl at pH 6.8, 2% SDS, 20% glycerol, 3% DTT, 0.04% bromophenol blue) containing the protease inhibitor cocktail (Roche, 04693132001) and phosphatase inhibitor cocktail (Roche, 04906845001). Samples were sonicated, boiled at 95 °C for 5 minutes and then centrifuged at 12,000 g for 10 min at 4°C. The supernatants were then loaded in 10% SDS-PAGE (Invitrogen) and probed with the primary and secondary antibodies listed below. The immunoblots were detected using the High-sig ECL Western Blotting Substrate (Tanon, e168230). Images were captured with an Amersham Imager 600 (GE Healthcare) and the densitometry was measured with ImageJ. The contrast and brightness were adjusted equally using Adobe Photoshop CC2019. GAPDH was used as a loading control for normalization as indicated in the figures.

Antibodies

The following primary antibodies were used in this study: rabbit anti-ERK (CST, 137F5), rabbit anti-pERK T202/Y204 (CST, 4370S), mouse anti-pTDP-43 (pSer409/Ser410) (Cosmo Bio USA, TIP-PTD-M01), rabbit anti-TDP43 (Proteintech, 10782-2-AP), mouse anti-GAPDH (Proteintech, 60004-1-Ig). The following HRP conjugated secondary antibodies were used: anti-mouse (Sigma-Aldrich, A4416) and anti-rabbit (Sigma-Aldrich, A9169). Given that the anti-ERK and the anti-pERK antibodies are both of the rabbit origin, to avoid the inaccurate quantification of the pERK/ERK ratios caused by incomplete stripping, equal amounts of the same samples were loaded parallelly on the same gel and subjected to western-blotting detection with the two antibodies separately.

RNA extraction and real-time quantitative PCR (qPCR)

For qPCR, total RNA was isolated from fly heads or dissected fly brains using TRIzol (Invitrogen, 15596018) according to the manufacturer’s instruction. After DNase (Promega, M6101) treatment to remove genomic DNA, the reverse transcription (RT) reactions were performed using All-in-One cDNA Synthesis SuperMix kit (Bimake, B24403). The cDNA was then used in real-time qPCR with the SYBR Select Master Mix (Life Technologies, 4472908) using the QuantStudio^{TM} 6 Flex Real-Time PCR system (Life Technologies). The mRNA levels of *actin* were used as an internal control to normalize the mRNA levels of genes of interest. The qPCR primers used in this study are listed below:

actin forward: 5’---GAGCGCGGTTACTCTTTCAC---3’

actin reverse: 5’--- GCCATCTCCTGCTCAAAGTC ---3’

Dsor1 forward: 5’--- CGACAATCACGCCATCATCC ---3’

Dsor1 reverse: 5’--- ACCAGCGACAGACCCAACG ---3’

rl forward: 5’--- TCATTCCGCAAACGTCTTGC---3’

rl reverse: 5’---AAGCCAGTATGATCGTGCTCG---3’

DptB forward: 5’--- ATCGTATGCCCAGCACCTT ---3’

DptB reverse: 5’--- TAGGCTTTGTCAACTGAATGGTA ---3’

AttC forward: 5’--- CAACACGCAGACCAAACCG ---3’

AttC reverse: 5’--- CTGGAAGCTATCCCGCACA ---3’

Dnr1 forward: 5’--- CATTGTCAACCTGCCCAAC ---3’

Dnr1 reverse: 5’--- GCGACAGACCTTCTCCAGAC ---3’

DptA forward: 5’--- CCACGAGATTGGACTGAATG ---3’

DptA reverse: 5’--- GGTGTAGGTGCTTCCCACTT ---3’

AttA forward: 5’--- GACACAATCTGGATGCCAAG ---3’

AttA reverse: 5’--- AATCCAGACCAGCTCCATTC ---3’

Drs forward: 5’--- CTGTCCTGATGCTGGTGGT ---3’

Drs reverse: 5’--- GCACAGGGACCCTTGTATCT ---3’

Dro forward: 5’--- TCGAGGATCACCTGACTCAA ---3’

Dro reverse: 5’--- ATGACTTCTCCGCGGTATG ---3’

Mtk forward: 5'--- CGTCGCCCTTCAATCCTA ---3'

Mtk reverse: 5'--- CGACATCAGCAGTGTGAATTT ---3'

Statistical analysis

Unless otherwise noted, the statistical significance in this study is determined by one-way ANOVA with Tukey's HSD post-hoc test, log-rank test (lifespan), or unpaired, two-tailed Student's *t*-test at *$p < 0.05$, **$p < 0.01$, and ***$p < 0.001$. The error bars represent the standard error of the mean (SEM).

SUPPLEMENTARY REFERENCES

1. Ni JQ, Markstein M, Binari R, Pfeiffer B, Liu LP, Villalta C, et al. Vector and parameters for targeted transgenic RNA interference in Drosophila melanogaster. Nat Methods. 2008;5(1):49-51.
2. Sun X, Duan Y, Qin C, Li JC, Duan G, Deng X, et al. Distinct multilevel misregulations of Parkin and PINK1 revealed in cell and animal models of TDP-43 proteinopathy. Cell Death Dis. 2018;9(10):953.

SUPPLEMENTARY FIGURES AND FIGURE LEGENDS

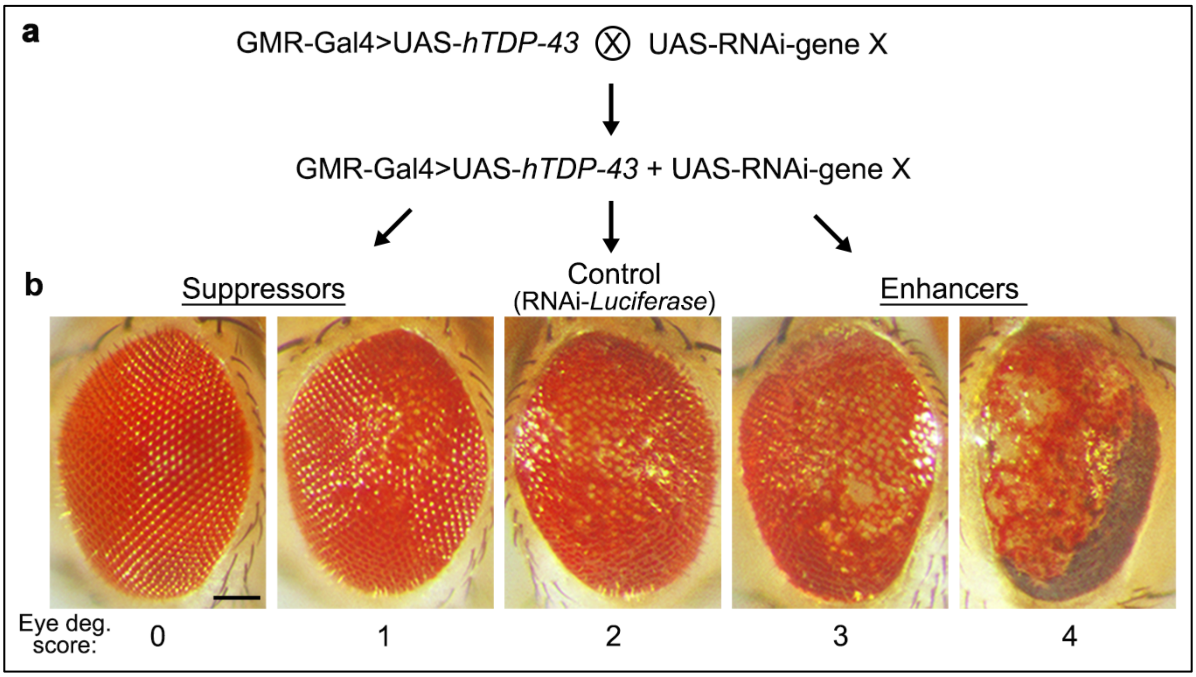

Figure S1. Screen for unknown genetic modifiers of TDP-43-induced cytotoxicity using the *Drosophila* eye as a model

(**a**) The scheme of the transgenic RNAi screen for new genetic modifiers of TDP-43 toxicity. (**b**) Examples of the external eye of the GMR>*hTDP-43* flies showing different levels of eye degeneration severity. Score 0: no deleterious change at all; Score 1: rough eye and less than 25% loss of pigment cells; Score 2: about 25%-50% loss of pigment cells; Score 3: about 50%-75% loss of pigment cells; Score 4: more than 75% loss of pigment cells or with obvious eye swelling and/or deformation. Intermediate scores were assigned when a sample appeared to fall between two classes. For example, score 0.5: only rough eye but no loss of pigment cells was observed. Deg., degeneration. Scale bar: 100 μm.

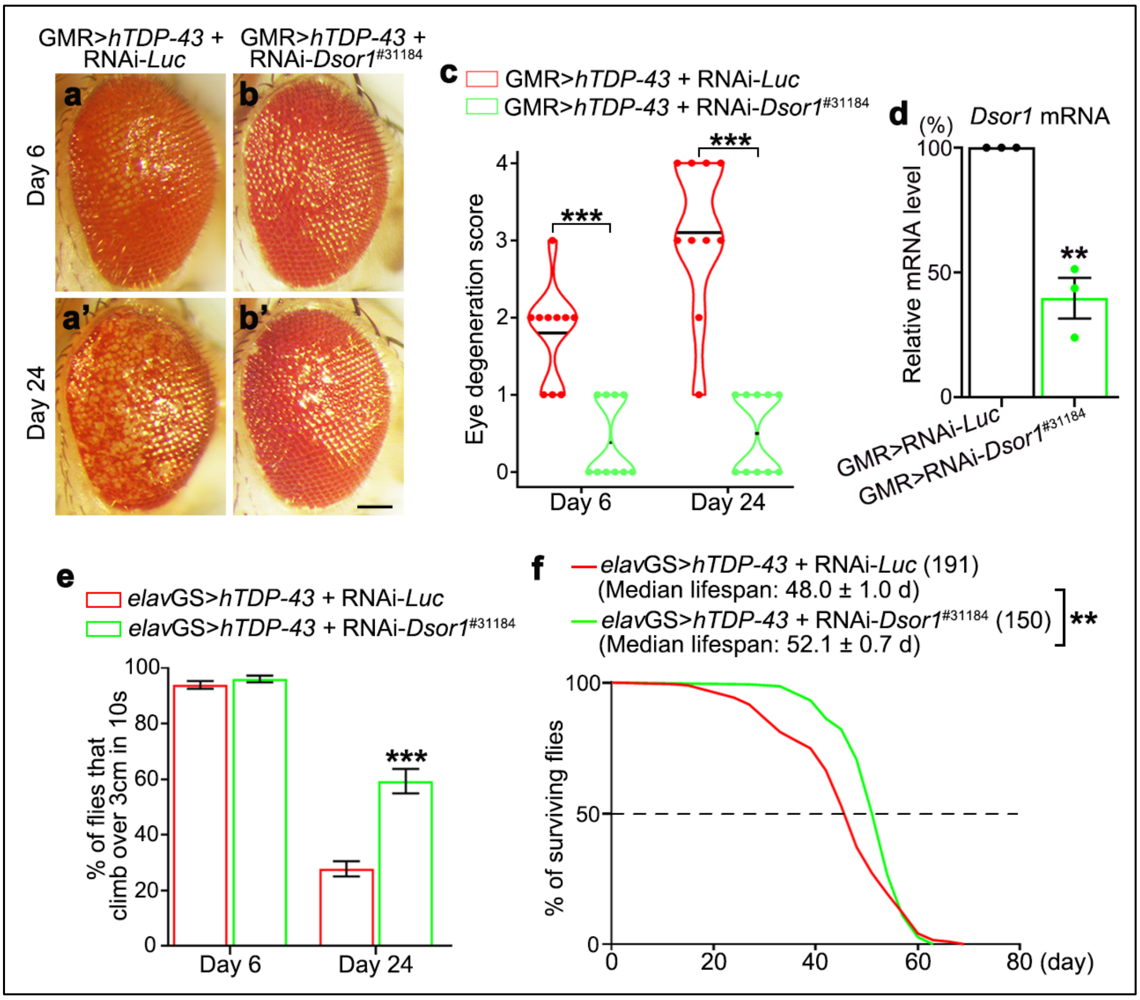

Figure S2. Another RNAi-*Dsor1* strain also suppresses the degenerative phenotypes of TDP-43 flies.

(**a-b'**) TDP-43-induced eye degeneration (by GMR-Gal4) is suppressed by another transgenic RNAi fly strain of *Dsor1* (#31184). (**c**) The degeneration scores in (a-b') are quantified and shown as violin plots with mean. (**d**) qPCR analysis confirming the downregulation of *Dsor1* mRNA levels, which are normalized to *actin* and shown as percentages to that of the control flies (set to 100%), RNAi-*Luciferase* (RNAi-*Luc*). Note that the RNAi-*Dsor1* transgene is expressed in the fly eye only (with GMR-Gal4), while the mRNA levels were examined in the homogenates of the entire fly head that includes many other cells expressing *Dsor1* but not RNAi-*Dsor1*. (**e**) Adult-onset neuronal expression of RNAi-*Dsor1* (#31184) suppresses TDP-43-induced, age-dependent climbing decline. (**f**) The log-rank analysis of the survival curves shows that KD of *Dsor1* by neuronal expression of RNAi-*Dsor1* (#31184) extends the shortened lifespan of the *elav*GS>*hTDP-43* flies. Mean ± SEM; n = ~10 eyes/group in (c), n = 3 (d), n = ~10 vials/group with ~20 flies each vial in (e), and the number (n) of flies tested in each group is as indicated in (f). Student's *t*-test; $**p < 0.01$ and $***p < 0.001$. Scale bar: 100 μm. See Table S1 for the exact genotypes in each of the fly assays in the figure; same below.

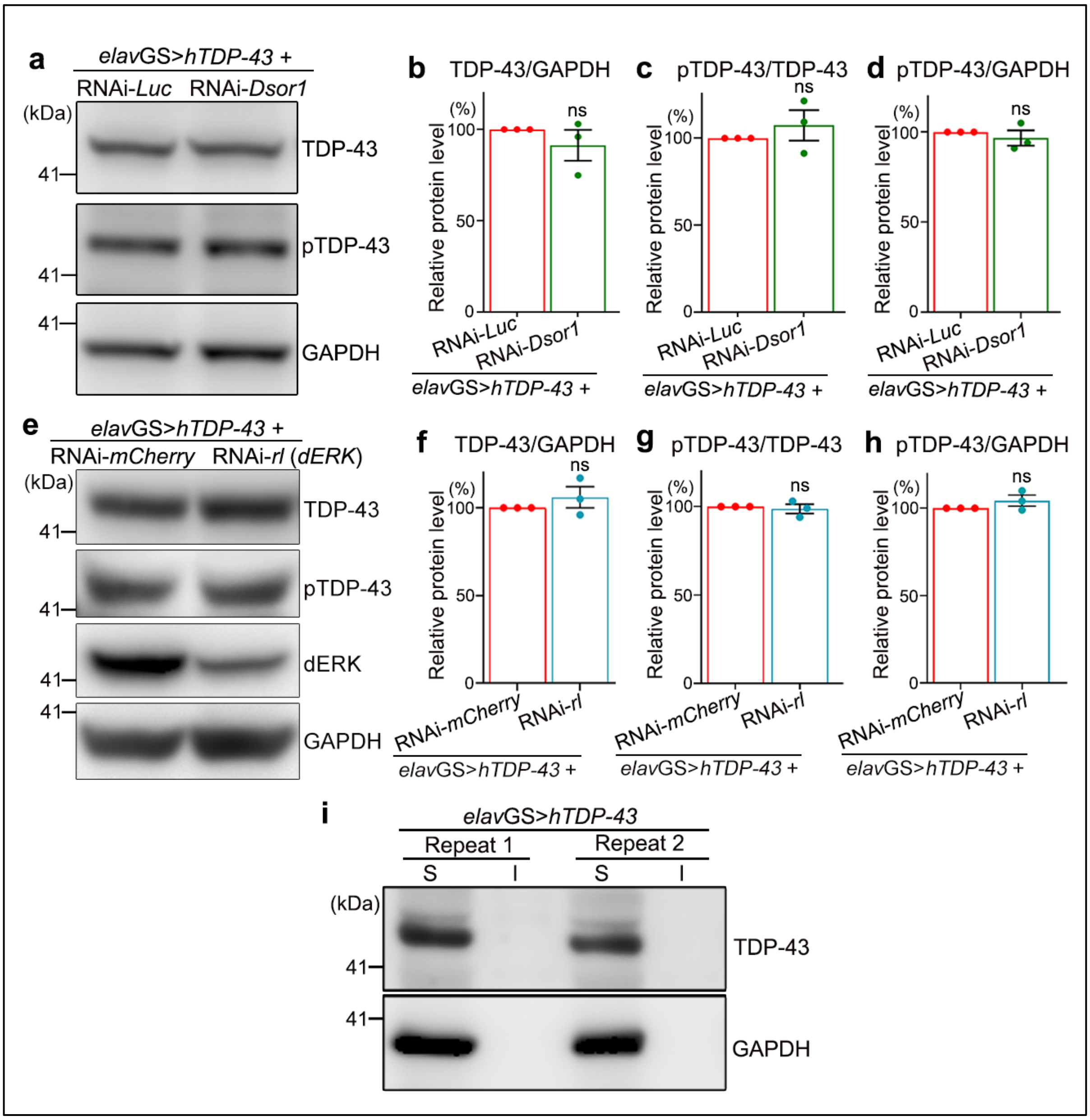

Figure S3. KD of *Dsor1* or *rl* does not reduce TDP-43 protein abundance or phosphorylation levels

(**a-h**) Representative western blot images (**a, e**) and quantifications of the relative protein levels of transgenically expressed human TDP-43 (**b, f**) and phosphorylated TDP-43 (pTDP-43) levels normalized to total TDP-43 (**c, g**) or GAPDH (**d, h**) in the "*elav*GS>*hTDP-43*+RNAi-*Dsor1*" (a-d) or "*elav*GS>*hTDP-43*+RNAi-*rl*" fly heads (e-f). Mean ± SEM; n = 3. Student's *t*-test; ns, no significance. (**i**) Western blot examination of transgenically expressed wild-type hTDP-43 protein in the soluble (S, supernatants in 0.1% SDS RIPA buffer) and insoluble (I, pellets resuspended in 9 M of urea) fractions of the fly head lysates. Two independent biological repeats are shown.

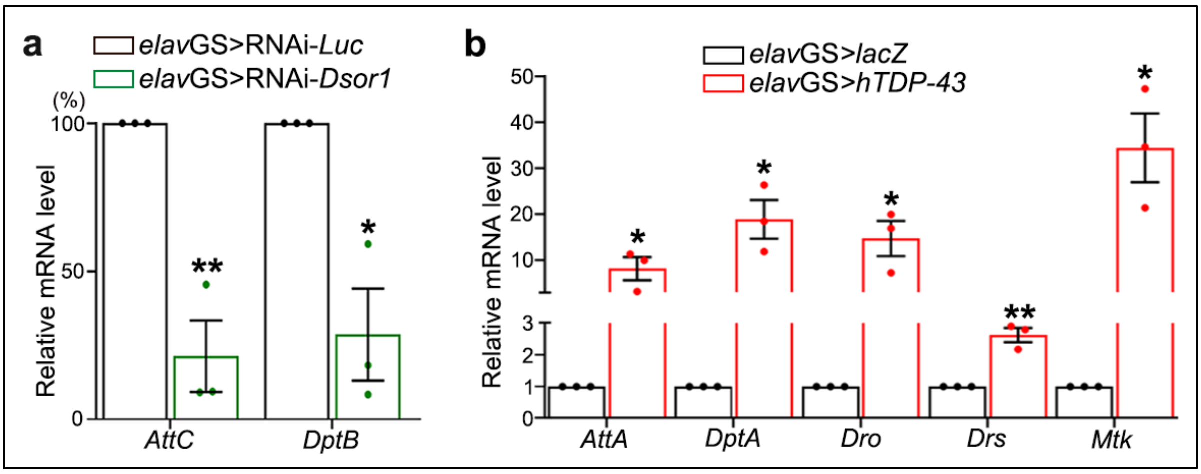

Figure S4. Examination of the immune AMP levels in fly heads with neuronal KD of *Dsor1* or transgenic expression of *hTDP-43*.

(**a**) qPCR analysis of the mRNA levels of the AMP genes *AttC* and *DptB* in the *elav*GS>RNAi-*Dsor1* fly heads. (**b**) qPCR examination of the mRNA levels of other immune AMPs in the *elav*GS>*hTDP-43* fly heads (in addition to Figure 3c). All mRNA levels are normalized to *actin* and shown as percentages (a) or fold changes (b) relative to that of the respective control flies (which is set to 1 or 100%): RNAi-*Luc* in (a) and UAS-*lacZ* in (b). Mean ± SEM; n = 3. Student's *t*-test; *$p < 0.05$ and **$p < 0.01$.

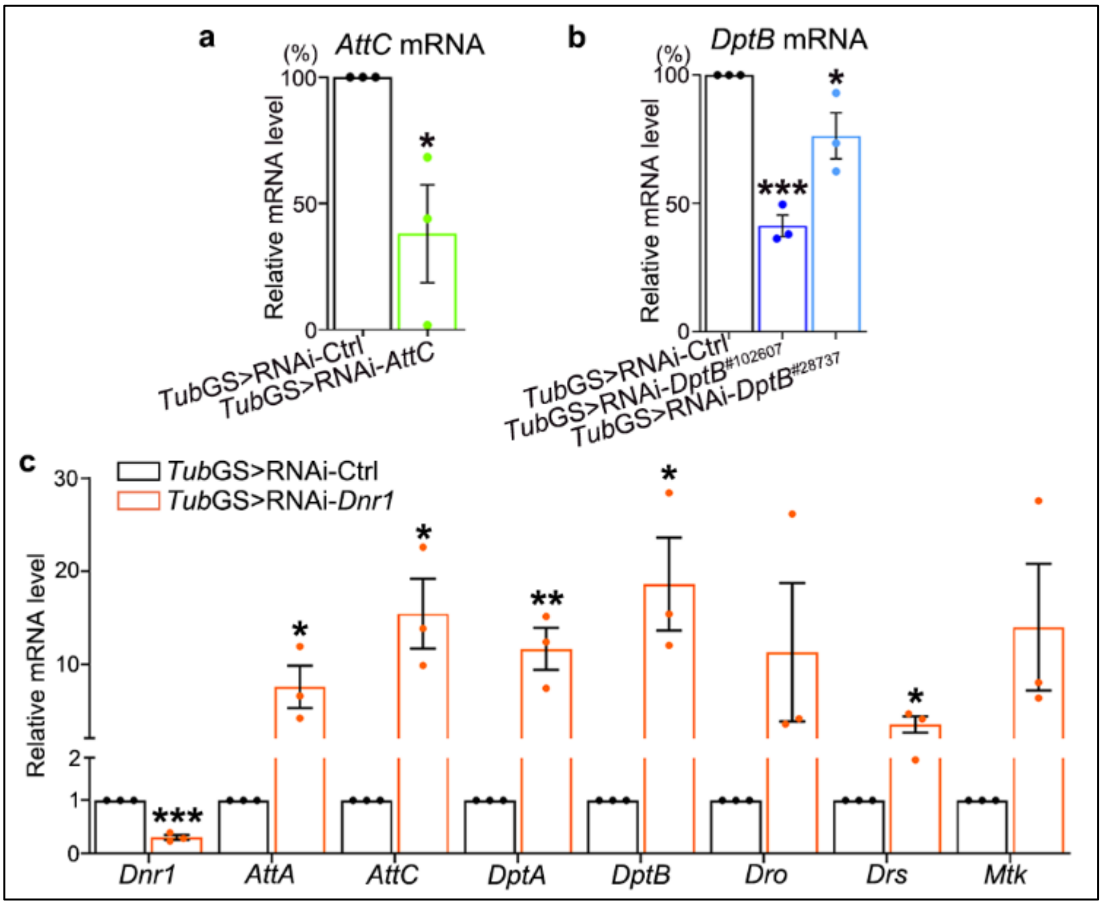

Figure S5. Examination of the AMP levels in fly heads with KD of *AttC*, *DptB* or *Dnr1*. (**a-b**) qPCR analysis confirming the downregulation of the mRNA levels of *AttC* and *DptB* in the *Tub*GS>RNAi-*AttC* (**a**) or *Tub*GS>RNAi-*DptB* flies (**b**). (**c**) The mRNA levels of the indicated AMPs in the *Tub*GS>RNAi-*Dnr1* fly heads are evaluated by qPCR analysis. The profound upregulation of immune AMPs in the RNAi-*Dnr1* indicates an immune overactivation. All mRNA levels are normalized to *actin* and shown as percentage (a-b) or fold change (c) to that of the control flies (*Tub*GS>RNAi-Ctrl^{V60200}), which is set to100% (a-b) or 1 (c). Mean ± SEM; n = 3. Student's *t*-test; *$p < 0.05$, **$p < 0.01$ and ***$p < 0.001$.

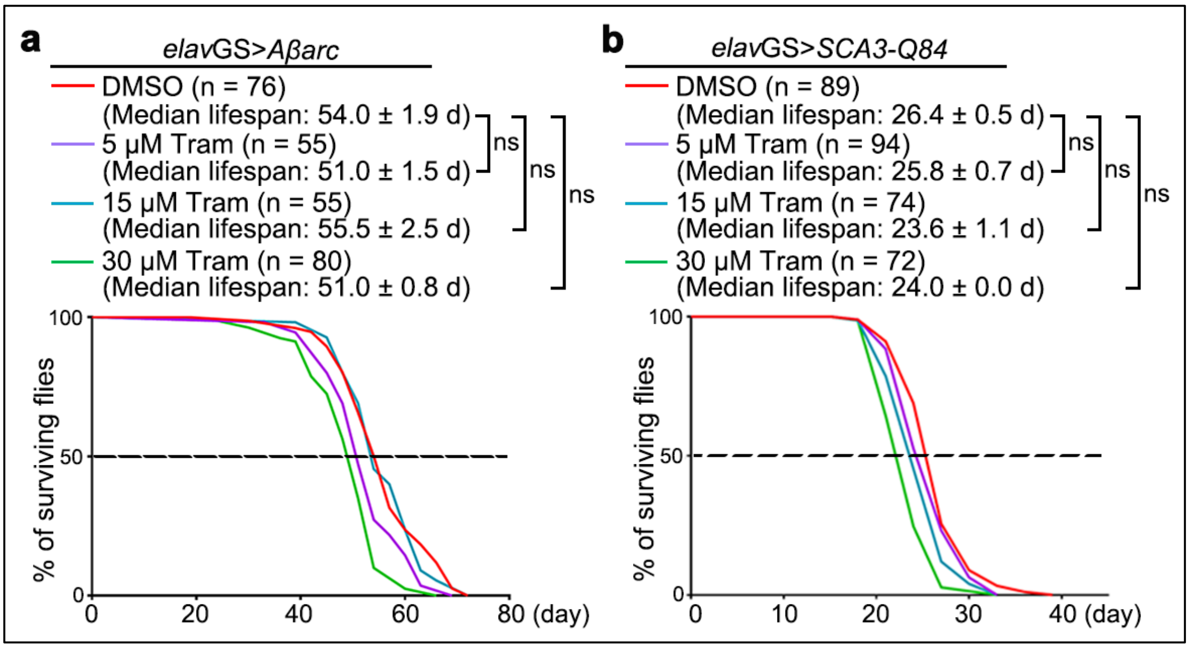

Figure S6. The effects of the MEKi Trametinib on the lifespan of the AD and SCA3 fly models.

The log-rank analysis of the survival curves indicates that trametinib does not extend the lifespan of the AD (*elav*GS> *Aβarc*) (**a**) or the SCA3 (*elav*GS>*SCA3-Q84*) (**b**) fly models. The number (n) of flies tested in each group is as indicated; the median lifespan is shown as mean ± SEM and the statistical significance is determined by one-way ANOVA. ns, no significance.

Supplementary Table 1. Summary of the specific genotypes of the flies used in each figure of this study.

See the separate datasheet file for Table S1.
